# Linking common mental disorders and asthma in Brazilian adolescents: a cross-sectional analysis of the ERICA study

**DOI:** 10.1016/j.jped.2025.01.003

**Published:** 2025-03-03

**Authors:** Mara Morelo Rocha Felix, Fábio Chigres Kuschnir, Érica Azevedo de Oliveira Costa Jordão, Bernardo Rangel Tura, Dirceu Solé, Maria Cristina Caetano Kuschnir

**Affiliations:** a Universidade Federal do Estado do Rio de Janeiro (UNIRIO), Departamento de Medicina Geral, Escola de Medicina e Cirurgia, Rio de Janeiro, State of Rio de Janeiro, Brazil; b Universidade do Estado do Rio de Janeiro (UERJ), Programa de Pós-graduação em Ciências Médicas (PGCM), Rio de Janeiro, State of Rio de Janeiro, Brazil; c Universidade do Estado do Rio de Janeiro (UERJ), Faculdade de Ciências Médicas, Departamento de Pediatria, Rio de Janeiro, State of Rio de Janeiro, Brazil; d Departamento de Pediatria, Escola de Medicina e Cirurgia, Universidade Federal do Estado do Rio de Janeiro (UNIRIO), Rio de Janeiro, State of Rio de Janeiro, Brazil; e Instituto Nacional de Cardiologia (INC), Departamento de Bioestatística e Bioinformática, Rio de Janeiro, State of Rio de Janeiro, Brazil; f Universidade Federal de São Paulo (UNIFESP), Departamento de Pediatria, Disciplina de Alergia, Imunologia Clínica e Reumatologia, São Paulo, State of São Paulo, Brazil; g Universidade do Estado do Rio de Janeiro (UERJ), Faculdade de Ciências Médicas, Núcleo de Estudos da Saúde do Adolescente (NESA), Rio de Janeiro, State of Rio de Janeiro, Brazil

**Keywords:** Asthma, Mental disorders, Adolescent, Smoking, Lifestyle, Risk factors

## Abstract

**Objective:**

Asthma is a heterogeneous chronic disease of the airways, affecting all age groups, especially children and adolescents. The aim of this study was to evaluate several factors associated with asthma in Brazilian adolescents.

**Methods:**

Cross-sectional study of a national representative sample of school-based adolescents aged 12–17 years from The Study of Cardiovascular Risk in Adolescents (ERICA) stratified by region and conglomerate by schools. The authors studied the following variables: sociodemographic characteristics, lifestyle, smoking, eating habits, sleeping and mental conditions.

**Results:**

Data from 66,567 adolescents were analyzed, 50.2 % of whom were female. Of the total, 52.7 % were between 12 and 14 years old. The overall prevalence of asthma was 14.5 % (95 %CI: 13.6–15.5). Asthma was associated with female sex (PR 1.35; 95 %CI: 1.15–1.57), white skin color (PR 1.25; 95 %CI: 1.04–1.50), private school (PR 1.26; 95 %CI: 1.05–1.52), smoking (PR 1.93; 95 %CI: 1.54–2.38); and alcohol consumption (PR 1.74; 95 %CI: 1.52–2.03). Excessive screen time (PR 1.19; 95 %CI 1.01–1.42) and short sleep duration (PR 1.28; 95 %CI 1.05–1.57) were also associated. Healthy eating habits, such as adolescents who ate breakfast, drank water, and ate meals with their parents, were associated with a lower prevalence of asthma. In relation to comorbidities, asthma was associated with common mental disorders (CMD) (PR 1.94; 95 %CI 1.64–2.27; *p* < 0,00001), but not with overweight or obesity (PR 1.09; 95 %CI 0.87–1.38). In the correspondence analysis, CMD was the strongest factor associated with asthma.

**Conclusion:**

Asthma was associated with several determinants in Brazilian adolescents, but the association with CMD deserves special attention in this age group.

## Introduction

Asthma is a chronic disease of the airways, generally inflammatory, characterized by bronchial hyperresponsiveness and variable obstruction of pulmonary airflow, leading to recurrent episodes of wheezing, dyspnea, and cough.^1^ It is considered the most common chronic non-communicable disease of childhood and adolescence, with an estimated prevalence of 300 million people worldwide.^2^

It is a complex multifactorial disease, related to genetic and environmental risk factors. There has been a rise in its prevalence, concomitantly with changes in lifestyle factors in the Western world.^2^ In adolescence, an increased risk for asthma morbidity and death is observed, because it is a period of physical and psychosocial changes, with more vulnerability among those with low socioeconomic status.^3^ Furthermore, there are age-related risk factors for asthma, such as smoking, alcohol consumption, mental health disorders, inadequate eating habits, and sedentary lifestyle.^3^^,^^4^ Obesity is also associated with asthma and its severity, especially in adolescent girls.^3^^,^^5^

The Study of Cardiovascular Risks in Adolescents (ERICA) was a national multicenter study that aimed to estimate the prevalence of cardiovascular risk factors, including obesity, diabetes mellitus (DM), systemic arterial hypertension, dyslipidemia, passive and active smoking, sedentary lifestyle, inadequate eating habits, and the association between these factors, in adolescents aged 12 to 17 years.^6^ This research, conducted in 2013 and 2014, also investigated the presence of asthma among ERICA participants, using a standardized and validated written questionnaire to estimate the prevalence of asthma in this population.^7^^,^^8^

In studies using data from ERICA, it was possible to demonstrate that the prevalence of asthma was significantly higher among female adolescents in Brazil.^9^ Furthermore, asthma was associated with smoking (passive and active) and short sleep duration.^10^^,^^11^ On the other hand, it was not associated with serum levels of vitamin D.^12^ In relation to metabolic parameters, it was observed that metabolic syndrome (MS) and some of its components were significantly associated with severe asthma in Brazilian adolescents.^13^

The objective of the present study was to evaluate several factors (demographic, socioeconomic, dietary, clinical, behavioral, mental, and environmental) and their relationships with asthma in Brazilian adolescents participating in the ERICA study.

## Methods

### Study design, population and data collection

Cross-sectional study using data from ERICA, a multicenter, school-based country-wide study performed in 2013 and 2014, in a complex sample of adolescents aged 12–17 years, enrolled in public and private schools.^6^ The study stratified the sample by region and grouped according to schools and classes with representativeness to the set of cities with >100,000 inhabitants of Brazil, all were state capitals.^6^ Detailed descriptions of subject recruitment and data collection have been reported previously.^6^ Data were collected by a self-administered questionnaire using a personal digital assistant (PDA). It contained approximately 100 questions divided into 11 sections: sociodemographic aspects, occupational activities, physical practices, eating habits, smoking habits, use of alcoholic beverages, reproductive health, oral health, referred morbidity, sleeping hours, and common mental disorders.^6^ Anthropometric measurements were performed with the student wearing light clothes and bare feet using an electronic scale (Líder® model P200 M –São Paulo, Brazil,) with a capacity of up to 200 kg and an accuracy of 50 g; height was measured using a portable stadiometer (Alturaexata® –Minas Gerais, Brazil) with an accuracy of 0.1 cm. Adolescents with physical disabilities who made the anthropometric assessment impossible and pregnant adolescents were excluded from the study.

### Measures

#### Block 1: Socioeconomic and demographic characteristics

*Demographic characteristics:* age (12–14 years, 15–17 years), sex (boys, girls).

*Socioeconomic conditions:* skin color (white or non-white), mother's educational level (no education or unfinished primary education, primary education or unfinished intermediate education, intermediate education or unfinished higher education, higher education), school's administrative status (private, public), school's location (capital, outside the capital) and possession of computers (no or yes - with or without internet access).

#### Block 2: Lifestyle factors

*Smoking:* the variables associated with smoking were defined as follows: ‘‘experimentation’’, adolescents who have smoked cigarettes at some point at least once in their lives; ‘‘current smoking (CS)’’, those who have smoked cigarettes on at least one day in the past 30 days; ‘‘regular smoking (RS)’’, those who smoked cigarettes for at least seven consecutive days in the past 30 days, and ‘‘passive smoking (PS)’’, adolescent non-smoker and had at least one smoker in the household.^14^

*Alcohol consumption:* this variable was defined by the question: *“*In the last 30 days (one month), on how many days did you have at least one glass or dose of alcohol?” Those who answered, “never drank alcohol” or “no day in the last 30 days” were classified as non-consumers of alcohol, and those who answered, “1 or 2 days”, “3 to 5 days”, “6 to 9 days”, “10 to 19 days”, “20 to 29 days”, or “every day” in the last 30 days were classified as consumers of alcohol.^15^ This classification was based on other national and international studies.^15^

*Sedentary lifestyle:* this variable was defined by the time in minutes of doing weekly physical activity, being classified, respectively, as sedentary (< 300 min/wk) and active (≥ 300 min/wk).^16^

*Screen time:* this variable was defined by the daily hours spent in front of screens, being classified, respectively, as adequate (≤ 2 h) and excessive (> 2 h).^17^

*Sleep duration:* the mean weekly duration of sleep was calculated according to the equation = (duration of sleep on the weekdays × 5) + (duration of sleep on the weekend days x 2)/7. “Short sleep duration” was defined as less than seven hours of sleep per night and “sufficient sleep duration” was defined as seven or more hours of sleep per night.^18^

*Eating habits:* the following eating habits were considered healthy: consuming breakfast, drinking water, and having meals with parents or legal guardians. The section about eating habits included questions at breakfast and on the company of parents or legal guardians during meals such as lunch and dinner, with the following answer options: “no”, “sometimes”, “almost every day” and “every day”. For the analysis, the authors grouped the responses to “almost every day” and “every day”, thus obtaining a variable with the options: “I do not consume it”; “sometimes I consume it” and “I consume it almost/every day”.^19^

#### Block 3: Health indicators

*Adiposity measures:* body mass index (BMI) was calculated using the following formula: BMI (kg/m^2^) = weight in kilograms divided by the height in meters squared. To determine the weight categories of adolescents, World Health Organization reference curves, with the index BMI/age, according to sex, were used. The cutoff points were as follows: very low weight, Z score less than −3; low weight, Z score −3 or more and less than −1; normal weight, Z score −1 or more and 1 or less; overweight, Z score >1 and 2 or less; obesity, Z score >2. Waist circumference (WC) was measured to the nearest 1 mm using a fiberglass tape. Measurement was done horizontally, at half the distance between iliac crest and lower costal margin.^20^

*Common mental disorders (CMD):* refer to two main diagnostic categories, depressive disorders and anxiety disorders, considered “common” due to their widespread prevalence in the population (around 20–30 %).^21^ The majority of CMD (about 90 %) are non-psychotic disorders (21). To measure this variable, the General Health Questionnaire, a 12-item version (GHQ-12) validated for the Brazilian population was used.^22^ The GHQ measures the mental health, especially psychiatric well-being, of an individual. Responses to individual items were coded as “absent” or “present” (0 or 1, respectively). Those with a score ≥ 3 were classified as cases.^21^^,^^22^

*Asthma:* was defined by the presence of at least one attack in response to the question: "In the last 12 months, how many attacks of wheezing have you had?". This question was part of the asthma module of the ISAAC standardized written questionnaire to estimate the prevalence of asthma in this population.^7^, ^8^, ^9^ Those who reported at least one wheezing attack in the last 12 months were diagnosed as having active asthma. Those who reported that they had “never had bouts of wheezing” or “no attacks in the last 12 months” were diagnosed as non-asthmatics. The presence of wheezing in the past 12 months shows high sensitivity and specificity (88.0 % and 90.0 %, respectively), compared to the evaluation of bronchial reactivity by provocation with methacholine, considered the gold standard for diagnosing asthma, according to a validation study performed in Brazil.^8^

### Statistical analysis

Primary sampling units and strata for the complex design of ERICA were considered for data analysis. Sampling weights, stratification, and clusters provided in the ERICA data set were incorporated into the analysis to obtain proper estimates. The authors performed a descriptive and inferential analysis of demographic (sex, age), socioeconomic (type of school, mother's education and computers at home), clinical (body mass index - BMI, abdominal circumference - WC), behavioral (smoking, alcohol use, sedentary lifestyle), mental health (common mental disorder - CMD), eating habits (intake of sweeteners, snacks, eating with parents) and environmental (location of school, geographic region) factors potentially linked to asthma.

Descriptive statistics were presented as frequencies and confidence intervals (CIs). Bivariate analyses between asthma and associated factors were conducted, estimating the prevalence ratio (PR) and its respective 95 % confidence intervals (95 % CIs) using Poisson regression with robust standard errors.

Additionally, a correspondence analysis was conducted to explore the relationships between asthma and other variables included in the study, serving as a basis for model definition. This multivariate statistical technique provides a visual representation of the associations between categorical variables on a graph, facilitating the identification of patterns and relationships within a large set of data. Following this, hierarchical cluster regression was performed to estimate the prevalence ratios between asthma and each block of variables, allowing for a stepwise evaluation of the contribution of different groups of factors to the outcome.

All analyses were performed using the SURVEY procedure in STATA 18.0 software (StataCorp, CollegeStation, TX, USA).

### Ethics

The ERICA study was conducted in accordance with the Declaration of Helsinki. It was approved by the Research Ethics Committee of the Instituto de Estudos de Saúde Coletiva of the Federal University of Rio de Janeiro (IESC/UFRJ) in 2009, and subsequently approved by the Ethics Committees of each of the 26 states and of the Federal District. Permission to conduct the study was obtained from all local and state Departments of Education.

Informed consent was obtained from participating students. When requested by the local Ethics Committee, informed consent was obtained from the parents. During data collection, students' privacy and confidentiality were maintained.

## Results

The authors analyzed data from 66,567 adolescents, 50.2 % of whom were female. Of the total, 52.7 % were between 12 and 14 years old. As for skin color, 43 % declared being white and 57 % non-white color (including black, mixed race, yellow, and indigenous). Table 1 presents the general characteristics of the participants according to their asthma diagnosis.

**Table 1:** Overall characteristics of the participants according to the diagnosis of asthma (ERICA, Brazil).

	Asthma		Without asthma	
	N	% (95 % CI)	N	% (95 % CI)
	8784	14.5 (13.6 −15.5)	57,783	85.5 (84.5–86.4)
Age				
12 - 14 years	3633	14.2 (12.9–15.6)	26,787	85.8 (84.4–87.1)
15 - 17 years	5151	14.9 (13.8–16.2)	30,996	85.1 (83.8–86.3)
Gender				
Female	5602	16.7 (15.5–17.9)	31,053	83.3 (82.1–84.5)
Male	3182	12.4 (11.4–13.5)	26,730	87.6 (86.5–88.6)
Skin color				
Non-white	4896	13.2 (12.4–14.1)	34,161	86.8 (86.0–87.6)
White	3739	16.5 (14.7–18.5)	22,089	83.5 (81.5–85.3)
School's administrative status				
Public	6576	13.9 (12.8–15.0)	45,684	86.11 (85.0–87.2)
Private	2208	17.5 (15.7–19.5)	12,099	82.5 (80.5–84.4)
Mother's education				
Unfinished primary education	1562	14.1 (11.4–17.4)	9976	85.9 (82.6–88.6)
Primary education	1342	14.5 (13.1–16.0)	8143	85.49 (84.0–86.9)
Secondary education	2512	16.8 (15.4–18.4)	14,610	83.17 (81.6–84.7)
College education	1854	16.4 (14.5–18.5)	11,385	83.61 (81.5–85.5)
Computers at home				
Yes	7382	15.1 (14.1–16.0)	46,623	84.95 (84.0–85.9)
No	1402	12.1 (10.4–14.2)	11,160	87.86 (85.8–89.6)
Current smoking				
No	8058	13.9 (13.0–14.9)	55,194	86.09 (85.1–87.0)
Yes	661	26,8 (23.0–31.0)	2228	73.24 (69.1–77.0)
Experimentation				
No	6408	21.4 (19.8–23.2)	47,672	87.0 (85.9–88.0)
Yes	2376	13.0 (12.0- 14.1)	10,111	78.6 (76.9–80.2)
Regular smoking				
No	8325	14,2 (13,2–15,2)	56,257	85.9 (84.8–86.8)
Yes	372	27.1 (23.1–31.4)	1084	72.9 (68.6–76.9)
Passive smoking				
No	6077	13.4 (12.2–14.5)	43,850	86.7 (85.5–87.8)
Yes	2707	17.7 (16.1–19.3)	13,933	82,4 (80.7–84.0)
Sedentary lifestyle				
Active	4209	15.0 (14.0–61.3)	27,084	85.0 (83.9–86.0)
Sedentary	3844	13.9 (12.5–15.3)	26,405	86.1 (84.7–87.5)
Screen time				
≤ 2 h/day	3138	13.5 (12.1–14.9)	23,053	86.5 (85.1–87.9)
> 2 h/day	4962	16.1 (15.0–17.2)	28,332	83.9 (82.8–85.0)
Sleep duration				
Sufficient sleep duration	7015	13.9 (12.9–15.0)	48,311	86.1 (85.0–87.1)
Short sleep duration	1769	17.8 (15.7–20.1)	9472	82.2 (80.0–84.3)
Alcohol consumption				
Non-consumers	5878	12.5 (11.7–13.3)	44,720	87.5 (86.7–88.3)
Consumers	2678	21.8 (20.1–23.7)	11,227	78.2 (76.3–79.9)
Meals at school				
Never	4222	14.9 (14.0–15.9)	27,056	85.1 (84.1–86.0)
Sometimes	2896	14.0 (12.3–15.9)	20,419	86.0 (84.1–87.7)
Always	1482	14.5 (12.7–16.4)	8830	85.5 (83.6–87.3)
Snacks in front of screen				
Never	696	11.4 (9.5–13.6)	6171	88.6 (86.4–90.5)
Sometimes	4353	14.4 (13.2–15.7)	29,542	85.6 (84.3–86.8)
Always	3551	15.5 (14.3–16.7)	20,592	84.5 (83.3–85.7)
Meals in front of screen				
Never	1169	14.1 (12.4–16.0)	7651	85.88 (84.0–87.6)
Sometimes	2426	13.1 (11.8–14.6)	17,620	86.87 (85.4–88.2)
Always	5005	15.4 (14.3–16.5)	31,034	84.62 (83.5–85.7)
Breakfast				
Never	2132	16.8 (15.2–18.4)	11,640	83.25 (81.6–84.8)
Sometimes	2684	15.2 (14.0–16.5)	16,169	84.79 (83.5–86.0)
Always	3784	13.1 (11.9–14.4)	28,496	86.89 (85.6–88.1)
Meals with parents				
Never	878	17.4 (15.3–19.7)	4317	82.63 (80.3–84.7)
Sometimes	2272	13.9 (12.6–15.3)	14,203	86.13 (84.7–87.5)
Always	5450	14.5 (13.3–15.7)	37,785	85.55 (84.3–86.7)
Drinking water				
Never	169	19.5 (14.9–25.0)	706	80.55 (75.0–85.1)
Sometimes	4359	15.6 (14.2–17.0)	25,780	84.44 (83.0–85.8)
Always	4072	13.3 (12.3–14.3)	29,819	86.71 (85.7–87.7)
Sweetener consumption^a^				
Never	6747	14.5 (13.4–15.7)	45,016	85.49 (84.3–86.6)
Sometimes	932	17.6 (15.6–19.8)	4966	82.37 (80.2–84.4)
Always	439	16.9 (13.4–21.2)	2315	83.07 (78.7–86.6)
Did not answer	482	9.7 (8.1–11.6)	4008	90.3 (88.4–92.0)
BMI				
Very low weight	20	10.7 (4.2–24.7)	218	89.3 (75.3–95.8)
Low weight	195	11.7 (9.3–14.6)	1521	88.3 (85.4–90.7)
Normal weight	6218	14.2 (13.1–15.4)	42,090	85.8 (84.6–86.9)
Overweight	1622	15.9 (14.2–17.8)	9566	84.1 (82.2–85.8)
Obesity	729	15.6 (13.4–18.1)	4388	84.4 (81.9–86.6)
Waist circumference				
Normal	7675	14.2 (13.3–15.2)	51,733	85.78 (84.8–86.7)
Elevated	1109	17.0 (15.1–19.2)	6050	82.96 (80.8–84.9)
CMD				
No	4597	11.4 (10.6–12.3)	41,281	88.58 (87.7–89.4)
Yes	4187	22.1 (20.2–24.17)	16,502	77.94 (75.9–79.8)

a High level of non-responders; BMI: body mass index; CMD: common mental disorders; 95 % CI (confidence interval).

The overall prevalence of asthma was 14.5 % (95 %CI: 13.6–15.5), being associated with female sex (PR 1.35; 95 %CI: 1.15–1.57), white skin color (PR 1.25; 95 %CI: 1.04–1.50), and private school (PR 1.26; 95 %CI: 1.05–1.52) (Table 2). The authors also observed a higher prevalence of asthma associated with mothers' higher level of education and having computers at home (Table 1).

**Table 2:** Prevalence ratios of determinants and health indicators in adolescents with and without asthma.

Characteristic	Prevalence ratio	95 % CI	*P*
Socioeconomic and demographic characteristics			
Age	1.05	0.89–1.25	0.66
Female sex	1.35	1.15–1.57	0.007
White skin color	1.25	1.04–1.50	0.039
Private school	1.26	1.05–1.52	0.024
Computers at home	1.25	0.99–1.54	0.046
Lifestyle factors			
Current smoking	1.93	1.54–2.38	< 0.001
Sedentary lifestyle	0.93	0.77–1.09	0.49
Elevated screen time	1.19	1.01–1.42	0.10
Short sleep duration	1.28	1.05–1.57	0.015
Alcohol consumption	1.74	1.52–2.03	< 0.00001
Health indicators			
Overweight	1.12	0.92–1.36	0.29
Obesity	1.09	0.87–1.38	0.38
Elevated waist circumference	1.20	0.99–1.44	0.08
CMD	1.94	1.64–2.27	< 0.00001

CMD, common mental disorders; 95 % CI, confidence interval.

The analysis of lifestyle factors showed that asthma was associated with smoking (PR 1.93; 95 %CI: 1.54–2.38) and alcohol consumption (PR 1.74; 95 %CI: 1.52–2.03) (Table 2). The prevalence of asthma was higher in the group with current smoking (26.8 %; 95 %CI: 23.0–31.0); regular smoking (27.1 %; 95 %CI: 23.1–31.4); passive smoking (17.7 %; 95 %CI: 16.1–19.3); and alcohol consumption (21.8; 95 %CI: 20.1–23.7) (Table 1). Excessive screen time (PR 1.19; 95 %CI 1.01–1.42) and short sleep duration (PR 1.28; 95 %CI 1.05–1.57) were also associated with asthma (Table 2).

Assessment of eating habits showed that healthy habits (adolescents who ate breakfast, drank water, and ate meals with their parents or legal guardians) were associated with a lower prevalence of asthma. The prevalence of asthma was higher in the group who “never eats breakfast” was 16.8 (95 %CI: 15.2–18.4); “never drinks water” was 19.5 (95 %CI: 14.9–25.0) and “never eats meals” with parents was 17.4 (95 %CI: 15.3–19.7) (Table 1).

Finally, the evaluation of health indicators indicated that asthma was associated with CMD (PR 1.94; 95 %CI 1.64–2.27; *p* < 0,00001), but not with overweight (PR 1.12; 95 %CI 0.92–1.36) or obesity (PR 1.09; 95 %CI 0.87–1.38) (Table 2).

Correspondence analysis was performed within each block (1,2 and 3) and then the blocks were unified in order to identify clusters of categories close to each other, indicating a strong association, or distant categories (weak association).

In the graphics below with the correspondence analysis (Figure 1A, Figure 1BA-Figure 1C), the authors observed that CMD was the strongest factor associated with asthma.

**Figure 1A:**
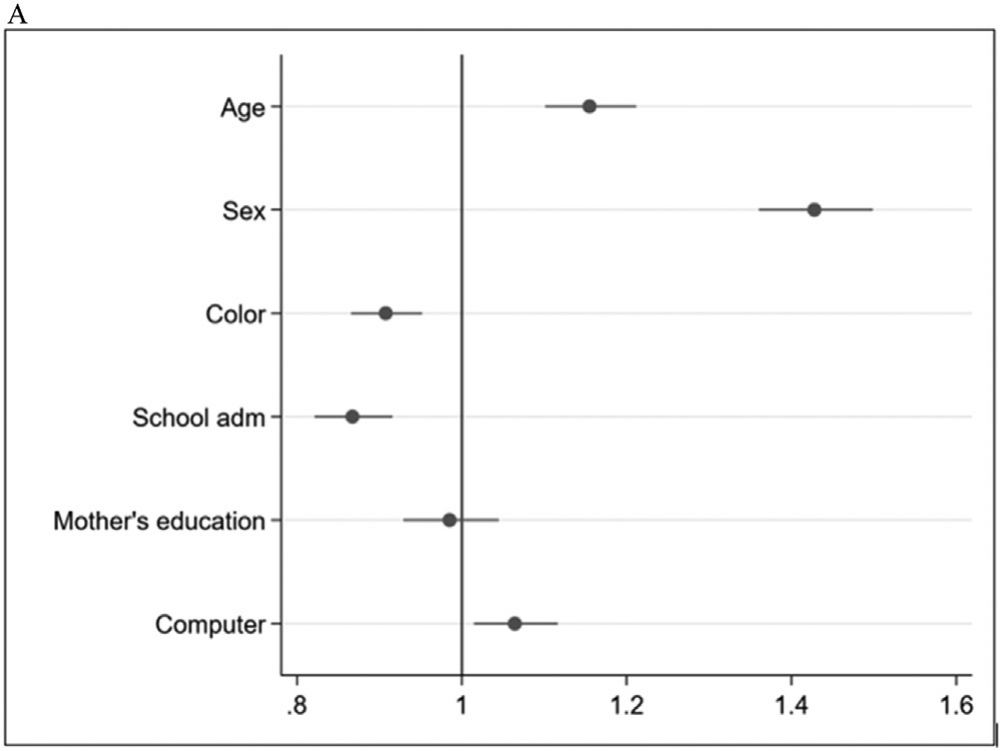
Correspondence analysis within block 1 (Fig. 1A), blocks 1 & 2 (Fig. 1B), and blocks 1, 2 & 3 (Fig. 1C). (1A) (Block 1 - Socioeconomic and demographic characteristics). Forest plot showing prevalence ratios (PR) and 95 % confidence intervals (95 % CI) for socioeconomic, and demographic factors (block 1) associated with asthma. Each point represents the PR for the corresponding variable, with horizontal lines indicating the 95 % CI. The vertical line at PR = 1 indicates no association. Variables with PR > 1 suggest a higher prevalence of asthma, while those with PR < 1 suggest a lower prevalence relative to the reference group.

**Figure 1B:**
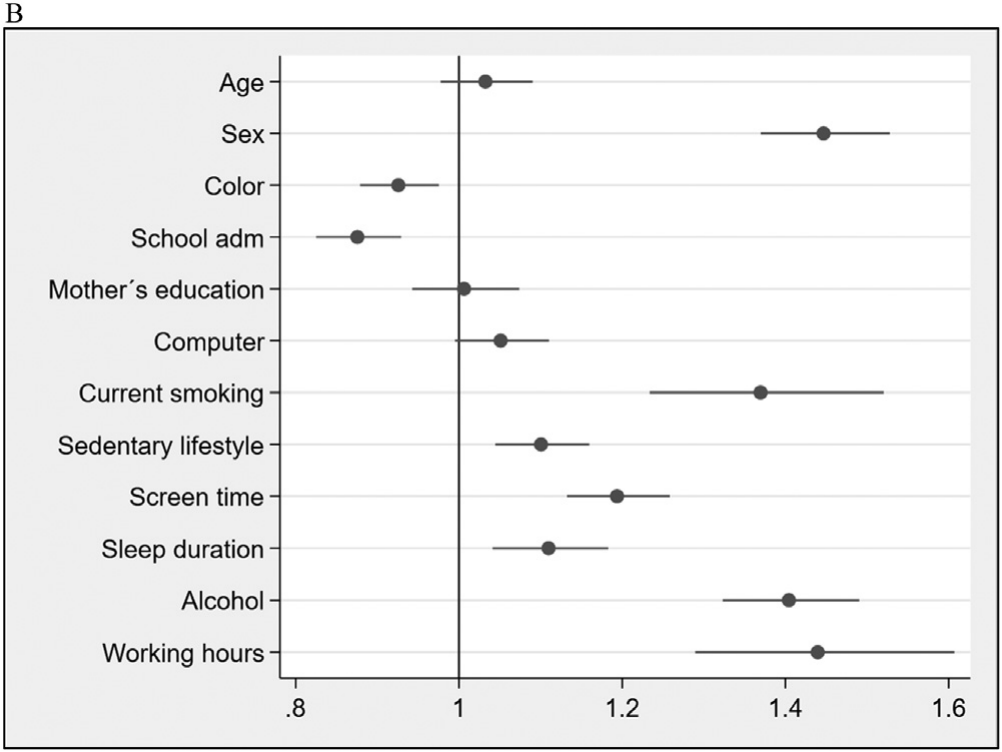
(Blocks 1 & 2-Socioeconomic / demographic characteristics & lifestyle factors). Forest plot showing prevalence ratios (PR) and 95 % confidence intervals (95 % CI) for socioeconomic, demographic, and lifestyle factors (blocks 1 & 2) associated with asthma. Each point represents the PR for the corresponding variable, with horizontal lines indicating the 95 % CI. The vertical line at PR = 1 indicates no association. Variables with PR > 1 suggest a higher prevalence of asthma, while those with PR < 1 suggest a lower prevalence relative to the reference group.

**Figure 1C:**
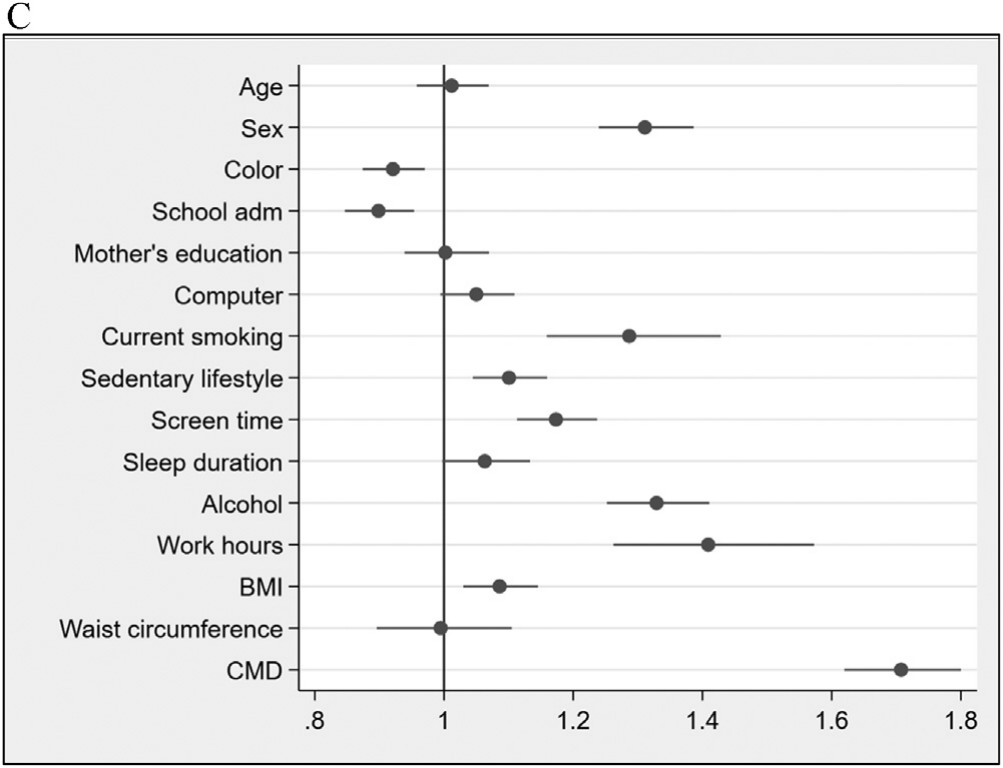
(Blocks 1, 2 & 3) –Socioeconomic / demographic characteristics, lifestyle factors & health indicators). Forest plot showing prevalence ratios (PR) and 95 % confidence intervals (95 % CI) for socioeconomic / demographic characteristics, lifestyle factors and health indicators (blocks 1, 2 & 3) associated with asthma. Each point represents the PR for the corresponding variable, with horizontal lines indicating the 95 % CI. The vertical line at PR = 1 indicates no association. Variables with PR > 1 suggest a higher prevalence of asthma, while those with PR < 1 suggest a lower prevalence relative to the reference group. BMI: body mass index; CMD: common mental disorders.

## Discussion

The present study identified several factors associated with asthma in Brazilian adolescents, including female sex, white skin color, higher socioeconomic status, lifestyle factors (such as smoking, alcohol consumption, excessive screen time, and short sleep duration), unhealthy eating habits, and certain comorbidities, particularly CMD. Among these, correspondence analysis revealed that CMD exhibited the strongest association with asthma.

Unlike studies that have focused on isolated variables, this research evaluated a wide range of factors and their relationships with asthma, providing a more comprehensive view of the determinants of asthma. The application of correspondence analysis allowed for the identification of the strongest associations, offering deeper insights into the relationships between asthma and other variables, particularly mental health. The study also explored factors associated with a lower prevalence of asthma, such as healthy eating habits.

Several studies have demonstrated the association between female sex and some lifestyle factors with asthma in adolescents. The PENSE-2012 Study was a cross-sectional health Brazilian survey with school children enrolled in the 9th year of elementary school. Similarly to the present results, they found that asthma was associated with the female sex, having smoked cigarettes, having tried alcoholic beverages, and some unhealthy eating habits, such as lunch or dinner time without the presence of parents or guardians, meals in front of the TV or while studying, and not having breakfast frequently.^23^

In a 5-year follow-up Korean study, the authors assessed risk factors for asthma among 15,481 adolescents (ages 12 to 15). They found that BMI, passive smoking, and living with a dog or cat, but not air pollution, were associated with an increased risk of wheezing.^24^ Another study from South Africa with 3957 adolescents demonstrated that severe asthma was associated with: fee-paying school quintile, overweight, exposure to traffic pollution, tobacco smoking, rhinoconjunctivitis, and eczema, all *p* < 0.01.^25^

Regarding psychosocial aspects, there is a growing number of studies evaluating these factors related to asthma in children and adults, but few in adolescents. However, some evidence suggests that adolescents exposed to stressors (e.g., poverty, exposure to violence, racism, and discrimination) and those who suffer from stress/anxiety or are depressed are at an increased risk of asthma.^26^

Investigators from the PENSE Study analyzed the relationship between social, environmental, and behavioral determinants and asthma symptoms among Brazilian students.^27^ They demonstrated that exposure to violence (feeling unsafe at school, being frequently bullied, being exposed to fights with firearms) and physical aggression by an adult in the family were the environmental factors with the strongest associations with asthma symptoms. For psychosocial indicators of mental health, feelings of loneliness and sleeping problems were the strongest factors, and among individual behavioral factors, the largest associations were found for tobacco consumption.^27^

In a longitudinal Australian study, the authors evaluated the association between asthma and anxiety. They found a unidirectional association between asthma in children aged 4–5 years and future anxiety development. Children with asthma (no anxiety at 4 years) had a higher prevalence of anxiety in adolescence compared with non-asthmatics.^28^

**Figure 2:**
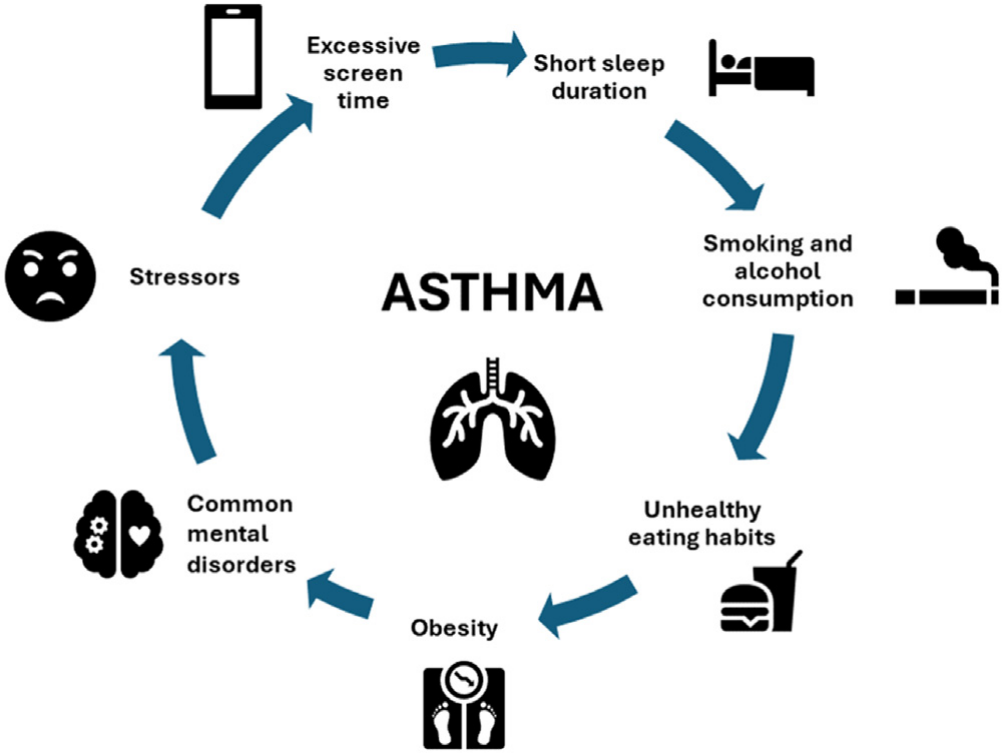
Relationship between asthma and behavioral factors.

Stress could also affect asthma through indirect mechanisms, including tobacco use, alcohol consumption, obesity, unhealthy eating habits, limited physical activity, and reduced adherence to treatment. There appears to be a bidirectional pathway in which stressors worsen asthma and poorly controlled asthma is associated with harmful behaviors that increase stress (Figure 2). Mental disorders are closely related to asthma, as the authors observed in the present study, in which CMD was the factor most strongly associated with asthma, but other determinants were also independently associated with asthma.

Genetic determinants may be involved in this relationship between asthma and mental disorders. A large-scale genome-wide cross-trait association study was conducted to investigate the genetic overlap between asthma from the UK Biobank and eight mental health disorders: attention deficit hyperactivity disorder (ADHD), anxiety disorder (ANX), autism spectrum disorder, bipolar disorder, eating disorder, major depressive disorder (MDD), post-traumatic stress disorder and schizophrenia. Cross-trait meta-analysis identified seven loci jointly associated with asthma and ADHD, one locus with asthma and ANX, and 10 loci with asthma and MDD.^29^

Another recent study evaluated genetic causal links between common mental disorders (specifically, anxiety and depression) and asthma. The authors found significant genetic correlations among sensations of anxiety or depression, MDD, and asthma.^30^ In bidirectional analyses, genetic liability to asthma was significantly associated with an increased risk of sensation of anxiety or depression and MDD. Conversely, genetic liability to anxiety disorders was not associated with an increased risk of asthma, nor was genetic liability to asthma associated with an increased risk of anxiety disorders.^30^

The present study has limitations. The cross-sectional design does not allow for establishing the temporality of relationships to infer causality between these factors and asthma. Furthermore, other factors correlated with asthma were not included, for example, family and personal history of atopy. The authors did not study the management and control of asthma, and we did not perform lung function tests.

On the other hand, this study also has strengths. The authors used standardized and validated procedures for measuring exposure and asthma variables. The authors evaluated a nationally representative sample of Brazilian adolescents and used a multidimensional approach, integrating socioeconomic, lifestyle, and behavioral factors alongside mental health, providing a better understanding of asthma's determinants.

In conclusion, asthma is a complex multifactorial disease influenced by various determinants, as observed in the sample of Brazilian adolescents. Notably, the authors identified a strong association between asthma and CMD, highlighting the importance of integrating mental health considerations into asthma management.

## Author contributions

Mara Morelo Rocha Felix: Conception and design of the study, analysis, and interpretation of data; drafting the article; revising it critically for important intellectual content; and final approval of the version to be submitted.

Fábio Chigres Kuschnir: Conception and design of the study, analysis, and interpretation of data; drafting the article; revising it critically for important intellectual content; and final approval of the version to be submitted.

Érica Azevedo de Oliveira Costa Jordão: Acquisition of data, analysis, and interpretation of data; revising it critically for important intellectual content; and final approval of the version to be submitted.

Bernardo Rangel Tura: Acquisition of data, analysis, and interpretation of data; revising it critically for important intellectual content; and final approval of the version to be submitted.

Dirceu Solé: Conception and design of the study; revising it critically for important intellectual content; and final approval of the version to be submitted.

Maria Cristina Caetano Kuschnir: Conception and design of the study; revising it critically for important intellectual content; and final approval of the version to be submitted.

## Conflicts of interest

The authors declare that they have no known competing financial interests or personal relationships that could have appeared to influence the work reported in this paper.
